# High Yield Overexpression, Refolding, Purification and Characterization of *Pseudomonas aeruginosa* Type B-Flagellin: An Improved Method Without Sonication

**Published:** 2016

**Authors:** Sobhan Faezi, Ahmad Reza Bahrmand, Mehdi Mahdavi, Seyed Davar Siadat, Iraj Nikokar, Soroush Sardari, Ian Alan Holder

**Affiliations:** 1 *Departments of Mycobacteriology and Pulmonary Research, Pasteur Institute of Iran, Tehran, Iran.*; 2 *Departments of Immunology, Pasteur Institute of Iran, Tehran, Iran.*; 3 *Laboratory of Microbiology and Immunology of Infectious Diseases, Paramedicine Faculty, Guilan University of Medical Sciences, Guilan, Iran.*; 4 *Biotechnology Research Center, Drug Design and Bioinformatics Group, Pasteur Institute of Iran, Tehran, Iran.*; 5 *Departments of Microbiology and Surgery, College of Medicine, University of Cincinnati, and Shriners Burns Institute, Cincinnati, Ohio, USA.*

**Keywords:** *Pseudomonas aeruginosa*, flagellin protein, pET28a, purification, vaccine

## Abstract

*Pseudomonas aeruginosa* as an opportunistic pathogen is a significant cause of acute and chronic infections in patients with compromised defenses. This bacterium is motile via a single polar flagellum made of polymerized flagellin subunits differentiated into two major serotypes: A and B. flagellin plays an important role as a virulence factor in the adhesion, colonization and invasion of *P. aeruginosa *into host epithelial cells. To develop a functional vaccine that can be used in practical application to prevent and treat infection, type B-flagellin was produced as recombinant protein. In this work, the *fliC* gene was introduced into a pET28a vector and expressed in *Escherichia coli* BL21 (DE3). The expressed recombinant protein was purified by a modified method without sonication using a HisTrap affinity column. The functional activities of produced flagellin were confirmed by ELISA, western blot analysis, motility inhibition assay and opsonophagocytosis test. The purification process of the type B-flagellin was lead to a high yield. The produced recombinant type B-flagellin showed high biological activity in all of these standard assays. In conclusions, this report provides the new protocol to efficiently obtain the type B-flagellin with high biological activity and immunogenicity. This immunogen can be introduced as an adjuvant or vaccine in the future study.

*Pseudomonas aeruginosa* (*P. aeruginosa*) is a common opportunistic human pathogen that is responsible for fatal infections in immunocompr-omised hosts and a frequent cause of acute pneumonia in patients who are being mechanically ventilated. More notoriously, this organism is also the main pathogen in cystic fibrosis patients and contribute to the chronic colonization of the lungs, leading to destruction and finally their death (1). In fact, *P. aeruginosa* infection rarely occurs in healthy individuals due to efficient clearance of the pathogen by the innate immune responses that include neutrophils and macrophages (2). This organism is motile via a single polar flagellum. This whip like appendage is required for swimming motility, chemotaxis, spreading in the environment, ability to translocate to preferred host cells, access to optimal colonization sites, and the Toll-like receptor 5 (TLR5)-dependent inflammatory respo-nse (3). TLR5 as a receptor has an important role in adaptive immune responses that recognizes specific pathogen-associated molecular patterns (PAMPs), which are conserved domains unique to microorga-nisms. By this mechanism, the flagellin activates macrophages, dendritic cells, and airway and corneal epithelial cells to produce proinflammatory mediators (4). The role of flagellum, as a virulence factor, in contributing to bacterial pathogenicity of many microorganisms, including *P. aeruginosa*, has been well established (5).

The flagellum as a complex organelle consists of a relatively complex basal body and hook structure attached to a filament. Flagellin is the major structural subunit of the flagellar filament that arranges itself in a hollow cylinder to form the filament. This boomerang-shaped protein comprises four major domains. The approximate 50 residues at the N and C-termini are responsible for polymerization and comprise D0 domain. The middle domain (D1) forms packed α-helical structures and contains highly conserved regions involved in TLR5 signaling. Smith et al. demonstrated that a short sequence of 10 amino acids (LQRIRDLALQ; amino acids 88 to 97) in the D1 domain of the flagellin of *P. aeruginosa* strain PAO1 was predicted to be important for binding to TLR5, leading to inflammatory responses (6). Domains D2 and D3 cover the hypervariable regions exposed as a folded β-sheet structure (7). On the other hand, the amino acid sequence alignment demonstrates that flagellin proteins are highly conserved in the N- and C-terminal regions, which correspond to D0 and D1 domains, and the variable regions form the outside surface-exposed domains (D2 and D3) in the assembled filament. Analysis of the crystal structure of *P. aeruginosa* flagellin suggests that D2 domain has a unique structure of two β-sheets and one α- helix that has not been found in other flagellins. It also has been suggested that the D2 domain would be exposed to solution and could play a key role in immuno-genicity (8).

Flagellin can be classified into two distinct serotypes, type A and type B based on antigenicity and apparent molecular weight of the subunits that are encoded by *fliC* gene. The B types consist of a homogeneous group with a flagellin molecular weight of 53 kDa whereas the heterogeneous A types can be divided into several subgroups comprising of flagellin molecular weight of 45-52 kDa. Although the two types of flagellin genes differ by 35% in primary structure, sequence alignments reveal a high homology between all *P. aeruginosa* flagellins. This evidence substantiates the intention to use flagellin as a vaccine against *P. aeruginosa* (9). It is presumed that the flagellin protein is an effective immunologic factor for immunity in *Pseudomonas* infections. The main objective of the present study was to express and purify the recombinant type B-flagellin (r-B-flagellin) from *P. aeruginosa* using a new purification procedure and evaluate its biological activity.

## Materials and methods

**Bacterial strains, and plasmids**

*Escherichia coli* (*E. coli*) strains BL21 (DE3) and Top10F, as expression and preservation hosts, were preserved in our laboratory. The widely studied strains of *P. aeruginosa* PAO1 and PAK were used as type B and type A flagellated strains. The type B- flagellin- encoded gene (*fliC *gene) was designed in the expression vector pET28a to produce the recombinant pET28a/*fliC*. This recombinant vector was synthesized by Biomatik Corporation (Cambridge, Ont., Canada). The strains were cultured in LB broth or on agar (Merck, Germany) at 37 °C with or without 30 µg kanamycin/ml (Bioscience, Canada).

**Construction of the expression vector**

The whole type B-flagellin gene (*fliC*) was inserted into the *E. coli* expression vector pET28a, in frame with a T7 promoter, kanamycin resistant gene and the C-terminal six-His-tagged sequence. The *fliC* gene containing a BamHI site located at the 5′ end, and a HindIII site located at the 3′ end. After the recombinant vector was transformed into *E. coli* Top10F competent cells, transformants were selected on LB plates (1% tryptone, 0.5% NaCl, 0.5% yeast extract, 1.5% agar, pH 7.5) supplemented with 30 µg/ml kanamycin. The recombinant plasmid pET28a/*fliC* was verified by polymerase chain reaction (PCR) and restriction enzyme digestion. The *fliC* gene was amplified by the colony PCR using the following oligonucleotides (Takapouzist, Iran) which have been designed for the *fliC* sequence of *P. aeruginosa* strain PAO1 from NCBI (GenBank accession no: NC_002516, Gene ID: 882052): 5'-CAT CAA CAG TGC CAA GGA CG-3' (forward) and 5'-GGC AGC GAA GTC GGT GTC-3' (reverse). The PCR program was initiated at 94 ºC for 4 min, followed by 30 cycles of 94 ºC for 45 s, 60 ºC for 1 min and 72 ºC for 1 min, and a final extension at 72 ºC for 10 min. The PCR products were sequenced for analysis of the accuracy of the sequence. The recombinant vector was also treated with the restriction endonucleases BamHI and HindIII (Jena Bioscience Kit, Germany) according to manufacturer's instruction. The PCR products and digested fragments were separated by 1.2% (w/v) agarose gel electrophoresis.

**Expression and isolation of inclusion bodies**

To over-express the protein, pET28a/*fliC *construct was transformed into BL21 (DE3) and platted on LB agar containing kanamycin (30 µg/ml). The colonies carrying pET28a*/fliC *were grown in 5 ml of LB medium supplemented with kanamycin at 37 ºC. At OD_600_ nm of 0.8, expression of the T7 promoter of the recombinant vector was induced by the addition of IPTG (Sigma, USA) at the early-exponential phase of growth to a final concentration of 1 mM. Following 21 h induction, cells were harvested and the induced level of r-B-flagellin was determined by 12% SDS-PAGE electrophoresis.

For isolation of inclusion bodies, an overnight culture of *Escherichia coli* BL21 (DE3) cells harboring pET28a/*fliC *was diluted 100-fold in LB medium (1 liter) containing kanamycin and incubated at 37 °C with shaking. When the OD_600_ of the culture reached 0.8, IPTG was added to a final concentration of 1 mM. After 21 h, the induced cells were harvested by centrifugation at 6500 g for 10 min at 4 ºC. A cell pellet obtained from a 1 L culture was resuspended in 50 ml lysis buffer (50 mM Tris-HCl pH 8.0, 300 mM NaCl, 20 mM β-mercaptoethanol (2-ME), 1 mM PMSF, 10 mM imidazole, 1% (v/v) Triton X-100) and incubated for 1 h at room temperature (RT) for proper lysis. The PMSF inhibit the proteolytic degradation of protein. Lysozyme lyophilized powder obtained from chicken egg white (BIO BASIC CANADA INC.) at a concentration of 0.1 mg/ml and DNase (0.01 mg/ml) were also added to the suspension and further incubated for 30 min at RT. The lysate was clarified by centrifugation (9000 g, 4 ºC, and 25 min). The pellets (approximately 3 g wet weight) that contained inclusion bodies (IBs) were resuspended in 50 ml of buffer A (20 mM sodium phosphate pH 8.0 and 300 mM NaCl). The insoluble fraction was collected by

centrifugation (9000 g for 40 min at 4 ºC).

**Solubilization, refolding and purification of r-B-flagellin**

The washed IBs were solubilized with guani-dinium buffer (20 mM sodium phosphate, 300 mM NaCl, 6 M guanidine hydrochloride, pH 7.4) for 1 h. The soluble and the insoluble fractions were separated by low speed centrifugation (4000 g for 7 min at 4 ºC). Both protein fractions of cell lysate were analyzed by 12% SDS-PAGE. The solubilized proteins were purified using Ni^2+^-NTA agarose (Qiagen, USA) according to the manufacturer’s instructions with modifications. Protein purification was carried out under hybrid conditions (denaturing and renaturing conditions) with modifications. Briefly, the solubilized IB was transferred into dialysis tubing of an appropriate MW cut-off (14000 MWCO) and dialyzed against denaturing binding buffer (8 M urea in buffer A, pH 7.4 ) for 6 h at 4 ºC under agitation. The dialyzed IB was loaded onto the pre-equilibrated column and then mixed with 2 ml Ni^2+^-NTA agarose resin for 1 h to allow the 6 × His-tagged protein to bind with Ni^+2^ in the column. The unbound contaminant proteins were washed by urea gradient solutions of refolding buffer (7 M to 0 M urea in buffer A, pH 6.0) along with 5% glycerol. The weakly bound contaminant proteins were washed three times away from the column using native wash buffer (20 mM imidazole in buffer A, pH 8.0). The bound r-B-flagellin proteins were eluted using a three-step gradient of imidazole (100, 250 and 500 mM) in buffer A, each step containing 8 ml of the respective buffer.

The eluted protein was transferred into dialysis tubing of an appropriate MW cut-off (14000 MWCO) and dialyzed against 1X PBS (pH 7.2) to remove imidazole and finally analyzed by 12% (w/v) SDS-PAGE followed by Coomassie brilliant blue R-250 staining. The protein concentration was quantitatively measured by using a NanoDrop 2000c spectrophotometer (Thermo Scientific, USA) and Bradford protein assay using

standard albumin (Sigma, USA).

**Immunoblot analysis**

The purified r-B-flagellin was electrophoresed in a 12% SDS–PAGE and then transferred onto PDVF membrane (Hi-bond Amersham Biosciences, USA) using a Mini-PROTEIN tetra cell (Bio-Rad, USA) at 100 mA for 1 h. The membrane was then blocked for 60 min in 5% (w/v) skim milk (Merck, Germany). After blocking, the primary antibody, rabbit polyclonal anti r-B-flagellin IgG, diluted 1:1000 in blocking buffer was added and allowed to incubate for 3 h at RT with shaking. After three times washing with TBST (Tris-buffered saline with 0.05 % Tween 20), the goat anti-rabbit IgG (H+L) secondary antibodies conjugated to HRP (Thermo Fisher Scientific) at a 1:2500 dilution in blocking buffer, was added and allowed to incubate for 1 h at RT with shaking. The membrane was then washed three times for 5 min each. Finally, it was developed by adding 3, 3′-diaminobenzidine (DAB) solution (Sigma, USA) allowing it to incubate until bands were seen. The reaction was stopped by rinsing with water. 

**Preparation and purification of polyclonal anti r-B-flagellin IgG**

Three months aged female New Zealand white rabbits (Pasteur Institute of Iran, Karaj, Iran) were immunized with 300 µg of the r-B-flagellin administered subcutaneously and boosted twice with 100 µg of purified r-B-flagellin with 2 weeks intervals. The rabbits were anesthetized intraperi-toneally (i.p.) with an injection of a mixture of ketamine (50 mg/kg) and xylazine (10 mg/kg). Blood samples were collected prior to immuni-zation and 2 weeks after each immunization. Approximately 20 ml of blood were collected in each step and incubated at 37 ºC for 2 h. Sera were collected from the retracted clot, and clarified by centrifugation (3500 g). Sera were aliquoted (1 ml) and stored at -70 °C. When sufficient r-B-flagellin antibodies were prepared, the rich fractions were pooled and purified using protein A/G agarose (Invitrogen, USA) according to the manufacturer's instructions. Protein concentr-ation in IgG fractions was quantitatively measured using a Bradford protein assay. Anti r-B-flagellin IgG and non-immune IgG were aliquoted at a concentration of 1–5 mg/ml and finally stored at -20 ºC until use. 

**Enzyme linked immunosorbent assay (ELISA)**

To detect the humoral immune responses against vaccine candidate, an optimized indirect ELISA was done. Briefly, 96-well plates (Nunc MaxiSorp) were coated with 0.5 μg of r-B-flagellin in carbonate coating buffer (pH 9.6) and incubated overnight at 4 ºC. The plate was then rinsed three times with PBS-T (140 mM NaCl, 2.7 mM KCl, 10 mM Na_2_HPO_4_, 1.8 mM KH_2_PO_4_, 0.05% Tween 20) and incubated with blocking solution (PBS containing 5% (w/v) skimmed milk) at 37 ºC for 2 h. After washing with PBS-T, a serial dilution (from 1:100 to 1:102400) from serum in blocking buffer was prepared and 100 µl of each one was added to each well. Following 2 h incubation at 37 ºC, plates were rinsed three times with PBS-T, and 1:10000- diluted HRP-labeled sheep anti-rabbit IgG was added to each well and incubated for 1 h at 37 ºC. After washing 5 times, the enzyme activity was determined by adding 100 µl of 3, 3, 5, 5’-tetramethyl benzidine (TMB) as substrate solution. Following incubation for 30 min, the reaction was stopped by adding 100 µl of 2N H_2_SO_4_ and optical density was read at 450 nm with an ELISA plate reader (Awareness Stat Fax 2100, USA).

**Motility inhibition assay**

To verify the functionality of the polyclonal antibody, three Petri dishes were filled with 10 ml of motility agar (LB with 0.3% (w/v) agar) which had been rehydrated with either; polyclonal or pre immune rabbit serum diluted 1:20. 20 µl of a cell suspension of *P. aeruginosa* strain PAO1 and PAK (OD_600_ = 0.2) in PBS was dispensed into the central well (5 mm in diameter) of each plate. For each assay, triplicate plates per serum were examined. The plates were incubated at 37 ºC. The mean diameters of bacterial spreading with sharp and less distorted rings were measured after incubation for 18 h (10). This experiment was carried out in triplicate.

**Opsonophagocytic killing assay**

The opsonophagocytosis assay was performed according to the method of Faezi et al*.* (11). Briefly, bacterial suspensions (PAO1 and PAK strains) were prepared at an approximate concentration of 2×10^9^ CFUs/ml in 1% bovine serum albumin (BSA). Mouse macrophages were used at a final concentration of 2×10^7^ CFUs/ml in RPMI 1640 supplemented with 10% heat-inactivated fetal bovine serum (FBS). A 3-week-old baby rabbit (Pasteur Institute of Iran, Karaj, Iran) was bled, and prepared sera were pooled and used as a complement source. Four different dilutions (1:4, 1:8, 1:16 and 1:32) of anti r-B-flagellin IgG were used. Complement activity of antisera was eliminated by heating at 56 ºC for 30 min. For the opsonophagocytic assay, the bacteria (2×10^9^ cells per well) were first incubated with an equal volume of diluted and heat-inactivated (at 56 ºC for 30 min) polyclonal IgG at 22 ºC for 60 min and then washed twice with BSA (1% (w/v)) for elimination of excessive antibodies. After suspending with 200 µl of 1% BSA, 100 µl of mouse macrophages was mixed with 100 µl complement in sterile 48-well microfuge plate (Greiner bio-one, Germany) and then incubated in a shaker at 37 ºC for 90 min. Shortly thereafter (time 0) and after 90 min, 25 µl of the mixture was removed, diluted in saline and finally plated for bacterial enumeration. Non-immune rabbit serum (NRS) (1:4 dilution) was used as pre-immune serum (control IgG). The opsonic killing activity of immune sera was compared to pre- immune sera statistically. Omitted antibodies, complement, or macrophage substituting with 100 µl of BSA were components of the control tubes. This experiment was performed in duplicate for each quantity. The following formula was used for the calculation of the percentage of killed bacteria in different groups, and the results were compared together.

Opsonophagocytosis (%)=(1-(CFU of immune serum/ CFU of pre -immune serum))×100

**Statistical analysis**

For the statistical analysis, SPSS 18.0 software was used. All data of this study were expressed as mean ± SD. The data were analyzed using one-way analysis of variance (ANOVA) and Student’s *t*- test (Statview). The p-values less than 0.05 were considered to be statistically significant.

## Results

**PCR amplification and construction of pET28a/ fliC**

**Fig. 1:**
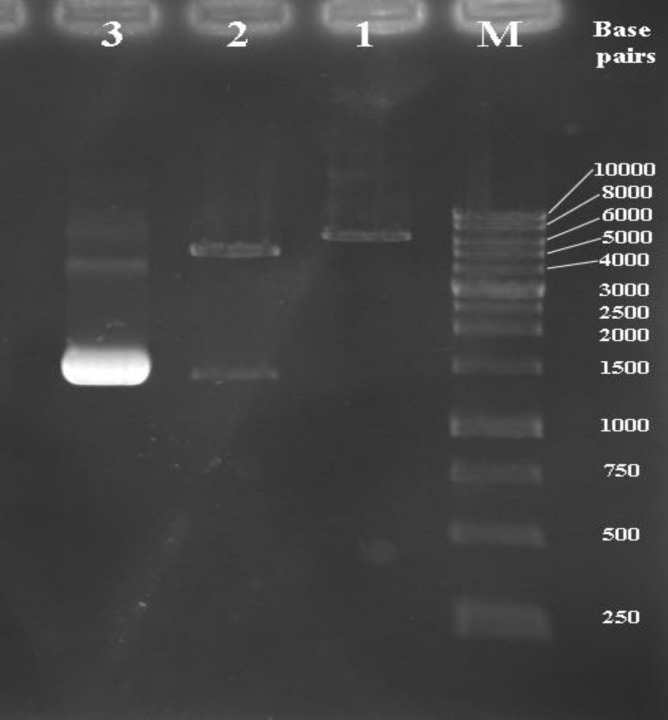
Agarose gel electrophoresis analysis of recombinant pET28a/*fliC* with restriction enzyme digestion. Lane M; DNA molecular weight marker (1 kb); lane 1: mono-digestion of the pET28a/*fliC* vector with BamHI. One expected fragment was observed on the gel (~ 6836 bp band). Lane 2: BamHI/HindIII digested the recombinant vector. Two expected fragments from double digestion were observed on the gel (~ 5369 and 1467 bp bands). Lane 3; the optimized PCR product of the *fliC* gene (~ 1467 bp band

The coding sequence of r-B-flagellin (*fliC* gene) was constructed in the pET28a expression vector. The *fliC* gene was flanked by BamHI and HindIII restriction sites at the 5′ end and 3′ end, respectively. Therefore, the coding sequence was preceded by a six His-tag at the N- terminal and a six His-tag at the C- terminal region of the gene. The construct was transformed into *E. coli* Top10F 

cells and selected on LB containing kanamycin. Transformants were characterized by colony PCR against specific primers. The target fragment of the* fliC* gene with the expected sizes is shown in Figure1. The recombinant vector pET28a*/ fliC* was extracted by Plasmid Mini Extraction Kit (BIONEER, Korea) and its orientation confirmed by digestion with BamHI and Hind III restriction enzymes. The two expected bands were observed on gel: 1467 and 5369 bp bands (Fig1, lane 2). Sequence analysis of recombinant pET28a/ *fliC* confirmed that there was no amplification errors and that construction was accurate.

**Overexpression and puriﬁcation of the r-B-flagellin**

**Fig. 2:**
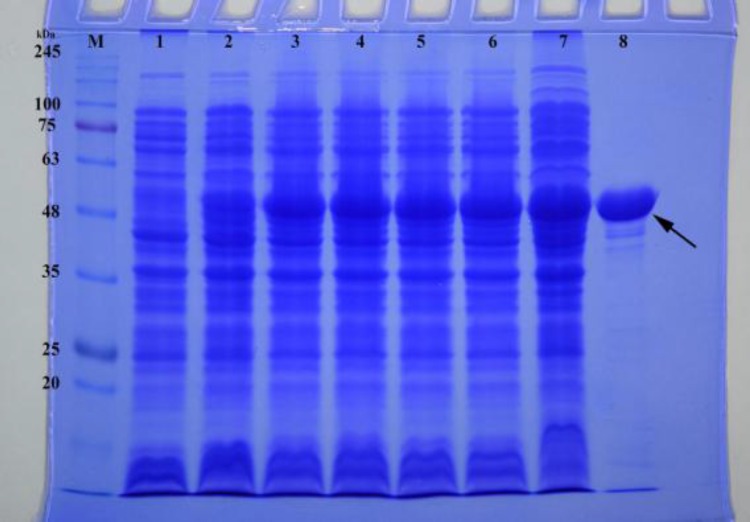
SDS-PAGE analysis of the expression of r-B-flagellin protein in *E. coli*. Lane M: molecular weight marker proteins; lanes 1-6: 1-6 h incubation after induction; lane 7: 21 h incubation after induction; lane 8: purified r-B-flagellin after HisTrap Chelating and Ni^2+^-affinity chromatography. The arrow indicates the position of the type B-flagellin (~ 53 kDa

To construct an overexpression system, the coding sequence of r-B-flagellin (*fliC *gene), whose theoretical molecular size is approximately 53 kDa, was constructed into expression vector pET28a to express a C- terminal His-tagged protein under the control of strong promoter T7 in *E. coli*. The recombinant plasmid, pET28a*/fliC*, was transformed into *E. coli* BL21 (DE3). This *E. coli *strain carries a chromosomal copy of the T7 RNA polymerase gene under control of the lacUV5 promoter. Addition of IPTG induces expression of T7 RNA polymerase resulting in transcription of the gene under control of the T7 promoter in cells harboring the pET28a/*fliC* vector. The SDS-PAGE analysis revealed that the highest amount of r-B-flagellin was produced by induction with 1 mM IPTG at 37 ºC for 21 h. The expression product of type B-flagellin protein was approximately 53 kDa in molecular size (Fig. 2). After stepwise dialyses, 8.54 mg renatured r-B-flagellin was obtained from 1 L of culture.

**Fig. 3:**
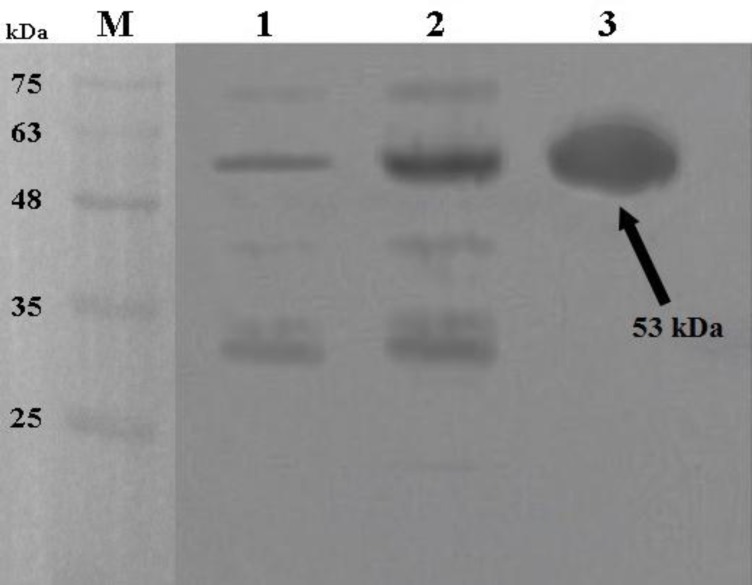
Western blot analysis of the expressed r-B-flagellin protein in *E. coli* BL21. Lane 1: total cell lysate of non-induced bacteria; lane 2: total cell lysate of bacteria after 21 h induction; lane 3: purified r-B-flagellin by Ni^2+^-NTA agarose; lane M: molecular weight marker proteins.

**Production of flagellin-specific IgG and specifi-city analysis**

Polyclonal antibodies against r-B-flagellin were produced in rabbit and finally the specific IgG was purified by using protein A/G aga-rose (Invitrogen, USA) according to manufac-turer's instruction. The checkerboard ELISA was performed to determine the suitable interac-tion between specific antiserum and r-B- flagellin. The anti r-B-flagellin IgG was detectable with 25600 times dilution of the original polyclonal antibody solution. However, 400 times dilution was used for the detection of flagellin for safety from the error. To determine the specificity of the antiserum against purified r-B-flagellin, immuno-blot analysis was performed. Non-induced and induced colonies and purified r-B-flagellin were immunoblotted and then hybridized with specific polyclonal IgG. A strong reaction was observed between specific antiserum and purified r-B-flagellin and somewhat between specific anti-serum and induced sample (Fig.3, lanes 2 and 3). A weak band was observed when the non- induced sample was probed with the antibody (Fig.3, lane 1). Overall, our results indicated that the rabbit-produced antibodies are highly specific to detect the r-B-flagellin.

**Motility inhibition assay**

**Fig. 4:**
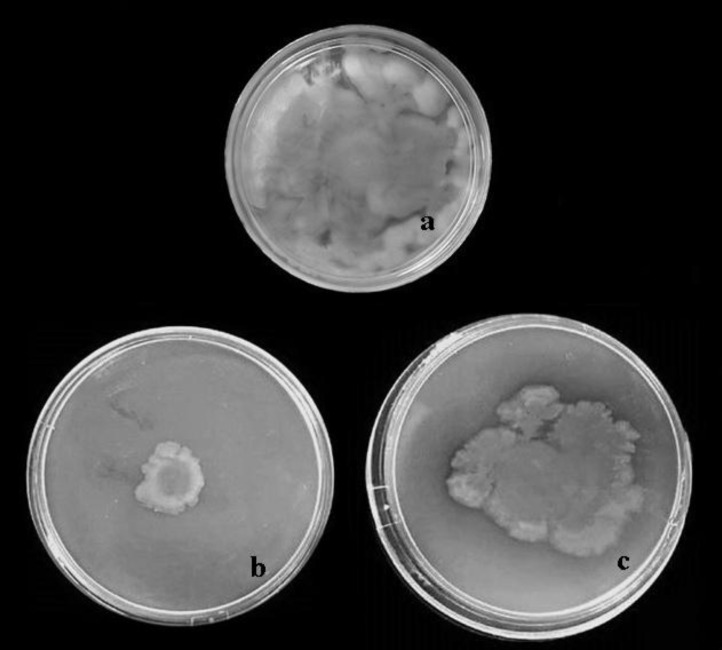
Motility inhibition of *P. aeruginosa* strains PA01 and PAK in the presence of anti r-B-flagellin IgG on motility agar. Antiserum raised against r-B-flagellin inhibited motility of *P. aeruginosa* PAO1 (b), with slight effects on strain PAK in motility agar (c) compared with non-immune rabbit serum (a

The biological activity of the immunized rabbit sera was tested in the motility inhibition assay for their functional activity to inhibit the motility of *P. aeruginosa* strains PAO1 and PAK strains. In this assay, non-immune rabbit serum (NRS) as non-immunized serum and PBS were used as controls. As shown in Table 1 and Figure 4, the specific anti r-B-flagellin IgG was able to dramatically inhibit the motility of the PAO1 at a dilution of 1:20 (B). The specific antiserum showed some cross reactivity to PAK as it slightly inhibited the motility of the strain (C). In the presence of NRS, no immobilization was observed (A).

**Table 1:** Motility inhibition of *P. aeruginosa* strains PA01 and PAK with antibodies raised against  r-B-flagellin

***P. aeruginosa*** **strains**	**Serum samples**	**Normal rabbit serum (NRS)**
	**Rabbit anti r-B-flagellin IgG **	
**PAO1**	22.97 ± 2.501 ***	98.63 ± 8.466
**PAK**	71.25 ± 1.120 &	96.27 ± 6.741

The mean diameters of bacterial spreading was measured according to millimeter (mean ± SD).

& *P* < 0.014;

*** *P* < 0.003

**Fig. 5:**
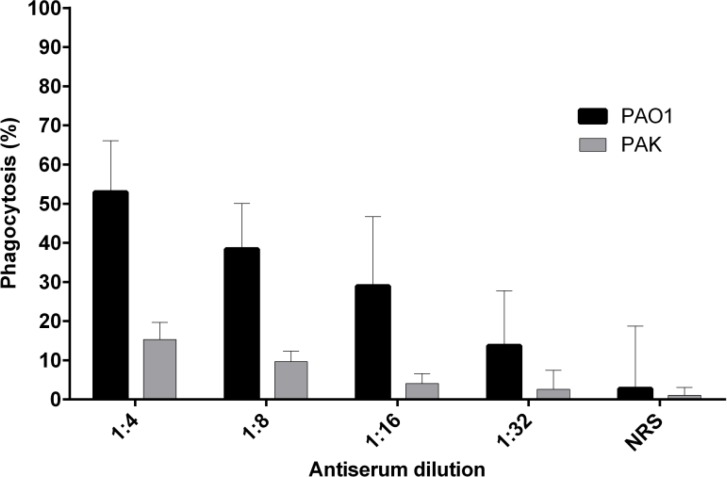
Phagocytic killing activity of rabbit polyclonal anti r-B-flagellin IgG against *P. aeruginosa* strains PA01 and PAK. When homologous strain PAO1 was treated with the antiserum to r-B-flagellin, considerable opsonic killing activity (52.99%) was observed compared to the control IgG (non-immune rabbit serum). Opsonic killing activity (15.3%) was also detected against heterologous strain PAK, but lower than PAO1. Bars represent means of duplicate determinations, and error bar indicates SD

**Opsonophagocytic killing activity**

To determine the bioactivity of anti r-B-flagellin IgG *in vitro*, its ability to promote phagocytosis of bacteria was evaluated by incubating *P. aeruginosa* PAO1 and PAK strains with pooled and diluted anti r-B-flagellin IgG and mouse macrophages in the presence of rabbit complement. In the presence of non-immune rabbit serum (NRS), as control group, only 2.75% opsonic killing activity was observed, which is most likely an indicator of non-opsonic phagocytosis. This study also showed that addition of anti r-B-flagellin IgG promoted phagocytosis of *P. aeruginosa* PAO1 strain and the number of viable bacterial cells decreased over 47% after 90 min as compared with the control group (Fig.5). When antibodies against r-B-flagellin protein were treated with the PAK strain, only 15.3% opsonic killing was detected. These data indicate that anti r-B-flagellin IgG act as an opsonin for killing the type B-flagellated strain (PAO1) but has a weak opsonic activity against the PAK strain of *P. aeruginosa*. 

## Discussion

Among the various recombinant immunodo-minant antigens identified as a candidate vaccine against *P. aeruginosa*, the type B-flagellin showed promising potential. Hence, we tried to improve the purification conditions to obtain high yield along with high level expression in pET28a vector system. In this study, the *P. aeruginosa* type B-flagellin gene (*fliC*) was designed into pET28a vector and was mainly expressed by *E. coli* strain BL21 (DE3) in the form of inclusion bodies (insoluble form). In the present study, two 6×His-tag was designed at the N-terminus and C-terminus of the r-B-flagellin coding sequence. His-tag, a small purification partner, has been designed into pET28a vector to decrease the time and cost of protein purification procedures without affecting protein well folding and bioactivity. The inclusion bodies that include whole type B-flagellin proteins were collected, purified and refolded. The pET system was chosen because it is a very powerful system developed specially for the cloning, expression and purification of recombinant proteins in *E. coli *and also has been utilized to overexpress exogenous proteins for decades.

To improve the purification conditions and to achieve higher yield of purified protein, modifications were made in the composition of lysis buffer. In this study, we have designed a lysis buffer that completely breaks down the bacterial cells so that after culture, no growth was observed after 24 h incubation. Most of the recombinant proteins though aggregate as inclusion body but can be solubilized and purified using hybrid condition. The significant increase in the yield of protein extraction from the inclusion bodies can be achieved by the addition of guanidine hydrochlo-ride (G-HCl) as a solubilization agent. In our study, purification of B-flagellin protein under hybrid conditions facilitates the purification in pET28a vector as the soluble fraction of protein. In this condition, the solubilized recombinant protein binds and washes under denaturing condition, and washes and elutes under native conditions. The use of 6 M G-HCl alone as strong denaturant was sufficient for the solubilization of protein from inclusion bodies. In this study, using optimum concentration of non-ionic detergent (Triton X-100) and reducing agent (β-mercaptoethanol) helped to efficiently purify the B-flagellin from inclusion bodies. The use of 1% Triton X-100 helps to improve the lysis conditions of cells for further solubilization of inclusion bodies. The optimal concentration of reducing agent (20 mM β-mercaptoethanol) also retains all cysteines in the reduced state and cleaves disulfide bonds that may have formed between contaminating proteins and 6×His-tagged protein. Furthermore, after three rounds of washes under native conditions, the proportion of irrelevant proteins was significantly decreased (data not shown). In addition, 5% (v/v) glycerol was added in the refolding buffer as it is reported as a refolding aid to enhance the stability and efficiently elevate the yield of proteins. After this, the solubilized inclusion bodies were equilibrated using dialysis against denaturing binding buffer. This process which improves the affinity purification, facilitates the binding of inclusion bodies to the matrix of the Ni^2+^-NTA agarose resin.

In recent years, high-throughput protein-refolding methods have been developed for the renaturation of inclusion bodies (12, 13). These include three methods such as dilution, dialysis or solid-phase separation for renaturation of inclusion bodies (14). In the present study, for the improve-ment of refolding process, we have selected dilution and dialysis methods. In on-column purification, we used a decreased gradient of urea for the gradual removal of urea and renaturation of recombinant protein. This washing process was followed by dialysis (with buffer exchange), in which there was no protein precipitation and aggregation. Our efforts at refolding of the solubilized proteins using dilution and dialysis methods led to effectively refolded desired recombinant protein. The modified purification protocol yielded 1.9-fold higher r-B-flagellin protein in pET28a as compared with conventional method. We found that after Ni-affinity chromatography, unrelated proteins further decreased, leading to an increase in the refolding yield and purity. In the present study, we did not only evaluates the efficiency of pET28a vector for the high level expression of the r-B-flagellin but also simultaneously developed a highly reprodu-cible and efficient procedure for purification and scalable production of the recombinant protein with high yield and high purity. The procedure developed here may be useful in the efficient purification of other recombinant proteins highly expressed in *E. coli* as inclusion bodies.

The immunoreactivity of purified r-B-flagellin under modified conditions was examined *in vitro* by ELISA, opsonophagocytosis and motility inhibition assays. The high titer of polyclonal antibodies was detected by ELISA method with purified r-B-flagellin, demonstrating the good immunogenicity of the protein and specificity of the antibody. In addition, our motility inhibition assay results showed that the antiserum raised against the r-B-flagellin could inhibit cell motility of *P. aeruginosa* PAO1 *in vitro*. The mechanism of this inhibition was not well understood. However, previous studies suggested that antibodies binding to the flagellar filaments cause bacterial agglutination and disruption of the rotational movements of the structure and lead to loss of colony spreading on the plates (15, 16). The opsonophagocytosis test results showed that anti r-B-flagellin had opsonic killing activity following treatment with homologous strain (PAO1), while the antiserum showed low opsonic killing activity against heterologous strain PAK. This outcome is contrary to the results of a study which determined that the anti type B-flagellin has no opsonic killing activity against either homologous strain PAO1 or heterologous strain PAK (17). Immunoblot analysis demonstrated that the specific polyclonal antibody could detect the recombinant protein expressed in prokaryotic cell (*E. coli* BL21). These findings indicate that the r-B-flagellin preserved correct folding. Since motility has been shown to be an important factor in microbial pathogenesis (18), therefore disruption of such a function by immobilizing antibodies *in vivo* may prove to be an advantageous prophylactic measure against pathogenic bacteria. These tests confirmed the bioactivity of the purified recombinant protein. Thus, the use of these reagents in the modified protocol does not have any adverse effects on bioactivity of the protein. We believe that, this is the first report on the expression and purification of whole type B-flagellin with a His-tag in bacterial system. In many studies, the protective efficacy of native flagellin as a vaccine has been proven in animals and humans (18-20). Since *P. aeruginosa* only contains two major antigenic types of flagellin, the immunoprotection of these can be evaluated as a bivalent vaccine or in combination with other antigenic substances in different mouse models of experimental infection *in vivo*. It was also suggested that the type B-flagellin could contribute as a vaccine or adjuvant to control *P. aeruginosa* infection.

In conclusion, the present study described an improved method for expression, purification and refolding of type B-flagellin of *P. aeruginosa* in *E. coli*. The recombinant proteins were expressed in the form of inclusion bodies under the pET28a vector. Here, we developed a reproducible and simplified method to achieve significantly yields of r-B-flagellin. The purification of r-B-flagellin was done under improved hybrid condition without sonication. In the vector, the higher amount of protein was expressed and formed inclusion bodies; however the conventional method of purification under denaturing conditions was not sufficient to recover protein completely. The procedure developed in this study might be useful in the efficient purification of other recombinant proteins expressed in *E. coli* as inclusion bodies. This recombinant protein was biologically active and recommended to be used as a vaccine or adjuvant.

## References

[1] Sadikot RT, Blackwell TS, Christman JW (2005). Pathogen-host interactions in Pseudomonas aeruginosa pneumonia. American journal of respiratory and critical care medicine.

[2] Lavoie EG, Wangdi T, Kazmierczak BI (2011). Innate immune responses to Pseudomonas aeruginosa infection. Microbes and infection / Institut Pasteur.

[3] McIsaac SM, Stadnyk AW, Lin TJ (2012). Toll-like receptors in the host defense against Pseudomonas aeruginosa respiratory infection and cystic fibrosis. Journal of leukocyte biology.

[4] Adamo R, Sokol S, Soong G (2004). Pseudomonas aeruginosa flagella activate airway epithelial cells through asialoGM1 and toll-like receptor 2 as well as toll-like receptor 5. American journal of respiratory cell and molecular biology.

[5] Haiko J, Westerlund-Wikstrom B (2013). The role of the bacterial flagellum in adhesion and virulence. Biology.

[6] Smith KD, Andersen-Nissen E, Hayashi F (2003). Toll-like receptor 5 recognizes a conserved site on flagellin required for protofilament formation and bacterial motility. Nature immunology.

[7] Honko AN, Mizel SB (2005). Effects of flagellin on innate and adaptive immunity. Immunologic research.

[8] Song WS, Yoon SI (2014). Crystal structure of FliC flagellin from Pseudomonas aeruginosa and its implication in TLR5 binding and formation of the flagellar filament. Biochemical and biophysical research communications.

[9] Miller WL, Matewish MJ, McNally DJ (2008). Flagellin glycosylation in Pseudomonas aeruginosa PAK requires the O-antigen biosynthesis enzyme WbpO. The Journal of biological chemistry.

[10] Faezi S, Sattari M, Mahdavi M (2011). Passive immunisation against Pseudomonas aeruginosa recombinant flagellin in an experimental model of burn wound sepsis. Burns : journal of the International Society for Burn Injuries.

[11] Faezi S, Safarloo M, Amirmozafari N (2014). Protective efficacy of Pseudomonas aeruginosa type-A flagellin in the murine burn wound model of infection. APMIS : acta pathologica, microbiologica, et immunologica Scandinavica.

[12] Tsumoto K, Ejima D, Kumagai I (2003). Practical considerations in refolding proteins from inclusion bodies. Protein expression and purification.

[13] Middelberg AP (2002). Preparative protein refolding. Trends in biotechnology.

[14] Singh SM, Panda AK (2005). Solubilization and refolding of bacterial inclusion body proteins. Journal of bioscience and bioengineering.

[15] Brett PJ, Mah DC, Woods DE (1994). Isolation and characterization of Pseudomonas pseudomallei flagellin proteins. Infection and immunity.

[16] Montie TC, Doyle-Huntzinger D, Craven RC (1982). Loss of virulence associated with absence of flagellum in an isogenic mutant of Pseudomonas aeruginosa in the burned-mouse model. Infection and immunity.

[17] Campodonico VL, Llosa NJ, Grout M (2010). Evaluation of flagella and flagellin of Pseudomonas aeruginosa as vaccines. Infection and immunity.

[18] Arora SK, Neely AN, Blair B (2005). Role of motility and flagellin glycosylation in the pathogenesis of Pseudomonas aeruginosa burn wound infections. Infection and immunity.

[19] Doring G, Meisner C, Stern M (2007). A double-blind randomized placebo-controlled phase III study of a Pseudomonas aeruginosa flagella vaccine in cystic fibrosis patients. Proceedings of the National Academy of Sciences of the United States of America.

[20] Holder IA, Naglich JG (1986). Experimental studies of the pathogenesis of infections due to Pseudomonas aeruginosa: immunization using divalent flagella preparations. The Journal of trauma.

